# Tribo-Mechanical Characterization of Carbon Fiber-Reinforced Cyanate Ester Resins Modified With Fillers

**DOI:** 10.3390/polym12081725

**Published:** 2020-07-31

**Authors:** Ankur Bajpai, Prateek Saxena, Klaus Kunze

**Affiliations:** 1 School of Engineering, Institute for Materials and Processes, The University of Edinburgh, Robert Stevenson Road, Scotland EH9 3FB, UK; 2 Manufacturing Department, Cranfield University, Bedfordshire MK43 0AL, UK; P.Saxena@cranfield.ac.uk; 3 Technische Universität Dresden, Institut für Leichtbau und Kunststofftechnik, 01062 Dresden, Germany; klaus.kunze@tu-dresden.de

**Keywords:** cyanate ester, tribology, carbon fibers, mechanical properties, fillers

## Abstract

High-performance polymer composites are being increasingly favored for structural applications. For this purpose, efforts are being focused on exploring the potential of high-performance thermoplastics and thermosets. Cyanate ester (CE) resin is a special thermoset that can be used at up to 400 °C without any considerable degradation; however, its tribological properties are not at the adequate level. Hence, it is needed to use this polymer in composite form with the fibrous/particulate reinforcement to impart better tribological properties and mechanical strength via a strong fiber–matrix interface. Carbon fiber/fabrics are at the forefront as reinforcement for specialty polymers. The tribological and tensile properties of cyanate ester (CE) composites-filled graphite, polytetrafluoroethylene (PTFE), and MoS_2_ micron-sized fillers reinforced with carbon fibers (CF) are investigated experimentally in a block-on-ring setup at 100 N, for 10 h, and with a sliding distance of approximately 10,000 m, against a hardened polished 100Cr6 steel shaft and diamond-like-coated (DLC) 100Cr6 steel shaft. The tribological properties of the composites including the coefficient of friction and specific wear rate are enhanced especially with the incorporation of graphite fillers. The friction coefficient and wear rate of the graphite-based composite was decreased significantly at 5 wt.% of graphite concentration. Further, at the same concentration, the graphite-based composite showed superior tensile properties as compared to the reference system owing to better dispersion and adhesion between the fibers and matrix. Tensile tests are performed to characterize the fiber–matrix interfacial adhesion and other strength properties.

## 1. Introduction

In recent years, owing to its strategic advantages, resin transfer molding (RTM) has attracted more and more interest in the production of advanced composites [1,2,3]. Particularly in comparison to several other composite material manufacturing methods, the main advantage of RTM is its flexibility to create a tailored matrix for the intended application. In order to be adequate for the RTM process, the matrix system must possess good functional properties, including low viscosity, appropriate injection temperature, and excellent reactivity. In recent times, a lot of research has gone into enhancing the performance of RTM matrix systems [4,5,6], but there is very limited research available for a matrix system that possesses a high glass transition temperature (T_g_) with a low injection temperature at the same time. Thus, cyanate ester (CE) resins are becoming more and more popular due to their ability to be injected at lower temperatures (<50 °C) and provide high T_g_ values [7].

CEs are presently appealing for electronic packaging and aerospace composite applications. This is because these thermosetting resins possess excellent mechanical properties, the ability to sustain high temperature, good adhesion, and low dielectric constant [8,9,10,11,12,13]. A major disadvantage associated with the CE resins is their brittle nature, which limits their application particularly as a friction material [14]. The brittleness is caused due to the presence of triazine groups, which have a high crosslinking density. Thus, the frictional and wear behavior of these resins are not good, and the toughening of the CE resins to make them suitable for industrial applications has become a topic of interest in recent years [15]. Several researchers have investigated and identified methods to enhance the tribo-behavior of CEs at high temperatures [14,16,17,18]. The primary techniques used to strengthen CE resins are combining them with other thermosetting resins [19,20], thermoplastics [21], nanoparticles [22], and certain elastomers [23,24]. Some of the researchers have also investigated and discussed the use of solid lubricant infiltration [25,26,27], but not enough literature is available on the assessment of their tribo-mechanical properties. Table 1 shows different coefficient of static friction values for certain types of materials against the specific counter-face material.

To improve the tribological properties and to open therewith applications in the area of high-temperature maintenance-free sliding bearings, it is required to modify those CE resins with special filler materials and/or fibers [28]. The addition of graphene oxide to the nanocomposite has a positive effect on the fracture toughness but, at the same time, the glass transition temperature is adversely affected [29]. Similarly, the mechanical properties can also be improved by adding MoS_2_ to the composite [30,31]. Improvement in the thermal stability and tribological properties by the addition of ZrB_2_ fillers was recently reported by Wu et al. [32]. 

The work done in this paper focuses on the infiltration of graphite, polytetrafluoroethylene (PTFE), and MoS_2_ fillers for the synthesis of CE-based carbon fiber-reinforced composites. The composites are developed using the resin transfer molding (RTM) technique. The mechanical strength and the tribo-properties of the composites are investigated, and the results are presented in this work.

## 2. Materials and Methods 

PT-30 (CE resin) supplied by Lonza chemicals is used as a matrix. PT-30 is a viscous liquid having a viscosity value of 400 mPa.s at 80 °C. Table 2 shows different properties of PT-30. Three different types of microparticles are used in this work: (a) PTFE micro powder (dyneon) with a size of 4 µm supplied by 3MTM, (b) graphite powder with an average particle size of 1.5 µm supplied by ALB materials, and (c) MoS_2_ (OKS-100) with an average particle size of 20 µm supplied by OKS Spezialschmierstoffe, GmbH. Carbon fiber laminates are manufactured as plates (300 mm × 300 mm × 3 mm), from which the required samples are machined. The plates are made of a 5 layer stack of Tenax IMS65 E23 (unidirectional fabric from Teijin, fiber diameter = 5 µm). A fiber volume fraction of 0.65 is used for all the composites.

**Figure 1:**
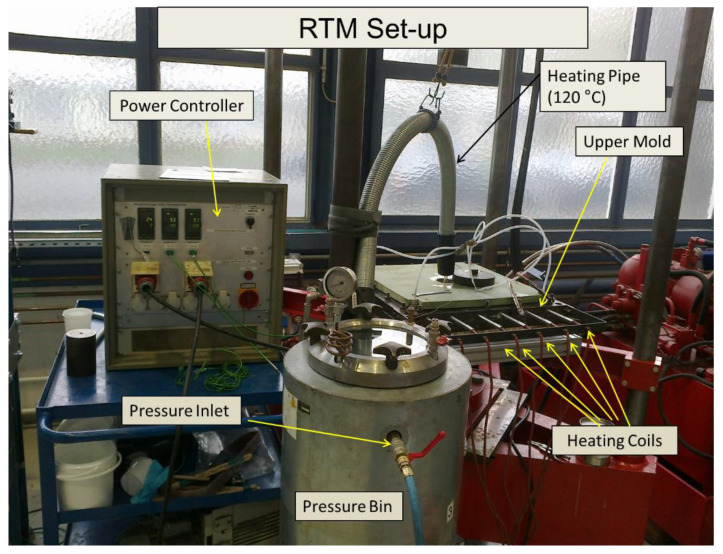
The resin transfer molding setup used for preparing different composites with labeled parts.

The composites are manufactured using the resin transfer molding (RTM) process, as shown in Figure 1. The major components of the RTM system are: An upper and lower mold, a cabin through which the pressure and temperature of the resin are controlled, a heating pipe that carries away the resin up to the injection port of the upper mold, a power supply, a control panel to maintain the desired level of temperature of the upper and lower molds, thermocouples, and a motor to evacuate the gases from the mold and pressure bin and other relevant accessories. The RTM process usually starts with the cleaning of the mold (upper and lower) with acetone. Once the cleaning is done, it is checked whether all the injection ports and vent ports are in a suitable position for the pouring of resin. If not, these ports are cleaned using a power-driven drill bit. The mold releaser solution (Mikon 305) is applied very properly in the lower and upper half of the mold to ensure the easy release of the mold once the whole process is finished. 

The mold plate is selected accordingly to the needs of the specimen. In our case, we have used the plate of dimensions (300 mm × 300 mm × 3 mm). The sealing of the mold is done with silicone rubber to avoid the pressure leak, with a pure resin plate, and adhesive tape is used to avoid the shrinkage of resin after the curing process. The upper part of the mold is put over the lower half very carefully through guiding screws; once it fits properly, tightening of the bolts is done (at 120 Nm). The thermocouple is connected on the upper and lower part of the mold to have a check on the difference between the input and output temperature. 

The resin is then put in a furnace at 120 °C for 1 h to attain the optimum viscosity of the PT-30 resin for injection. The temperature of the heating pipe fixed at 120 °C, which carries resin to the injection port. Then, the resin is taken out from the furnace and put into the pressure bin, which is already maintained at the temperature of 120 °C; then, the cover of the pressure bin is closed, and the bolts are tightened. A motor is attached to the pressure bin whose function is to suck out the air from the mold, pressure bin, and pipes, which ultimately creates a negative pressure in the system. This process is carried out for 10 min. The pressure is now slowly released, allowing the atmospheric pressure to get into the pressure bin, thereby creating a pressure difference in the mold and pressure bin. This results in a smooth flow of the resin from the pressure bin to the mold. Once the atmospheric pressure is reached in the whole system, pressure is applied to the pressure bin from an external source, which accelerates the flow of resin to the mold from the container in the pressure bin. The resin goes in the mold until it starts coming out from the vent ports of the mold, which gives an indication that the mold is completely filled with the resin. The pipes attached to the vent ports are clipped with special clippers, which restrict the flow of resin from the vent ports to ensure the proper filling of resin in the mold. After this step, the program is run from the controller for proper curing of the PT-30 resin.

**Figure 2:**
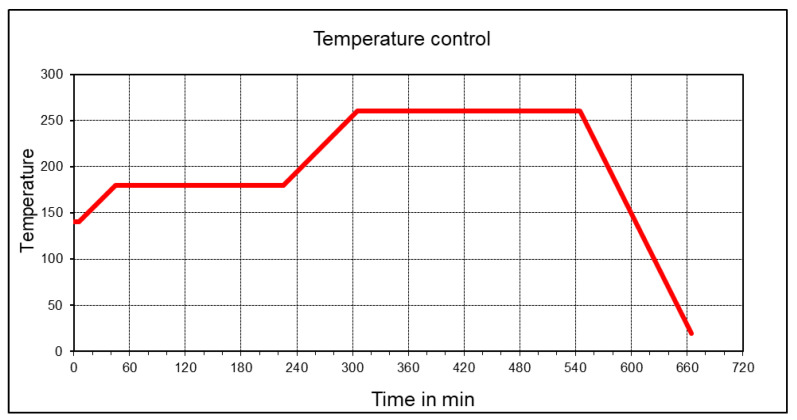
Typical curing cycle for PT-30 resin.

In the present work, no accelerator is used, as cyanate ester copolymerizes at 260 °C. The run-time for the RTM setup was nearly 11 h, as shown in Figure 2. For composites with microparticles, the required amount of microparticles is mixed with CE resin at 100 °C in Dispermat, Getzmann GmbH, Reichshof, Germany at 300 rpm for 1 h. Later, this mixture is used in the RTM process for preparing composites with carbon fiber.

Four different types of composites are prepared from the RTM process:

1. PT-30/carbon fiber (CF)
2. PT-30/CF/5 wt.% PTFE plate
3. PT-30/CF/5 wt.% MoS_2_ plate
4. PT-30/CF/5 wt.% graphite plate

### 2.1. Mechanical Properties

**Figure 3:**
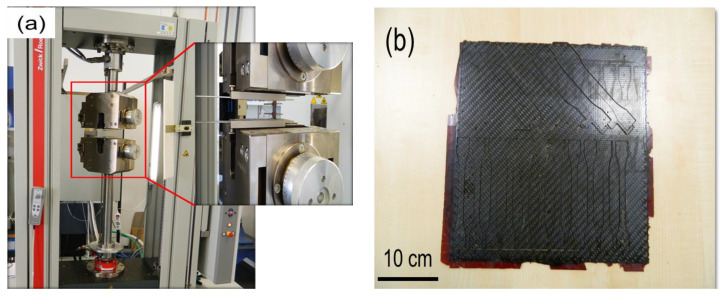
(**a**) The Universal tensile testing setup used to measure the tensile properties of prepared composites. (**b**) carbon fiber (CF)/PT30 composite plate with dog bone samples cut from abrasive water jet machine.

Tensile testing of the composites is carried out on a Zwick Roell Z100 as per ISO 527-4 at 23 °C (refer Figure 3a). Stress is applied by electrical rotation of a vertical screw onto which the sample-holding carriage is mounted. Electrical strain gauges measure the elongation of the sample and the output is directed to a controlling PC, which both controls and records the test values in real-time. The specimens from the composite plates are cut by using an abrasive jet water cutting machine. The cutting plan was drawn on a scale of 300 mm × 300 mm in the AutoCAD software package. This machine uses a jet of water at high velocity mixed with abrasive to cut the composite plates. From each composite plate, a total of 10 specimens are cut (Figure 3b).

### 2.2. Tribological Tests

**Figure 4:**
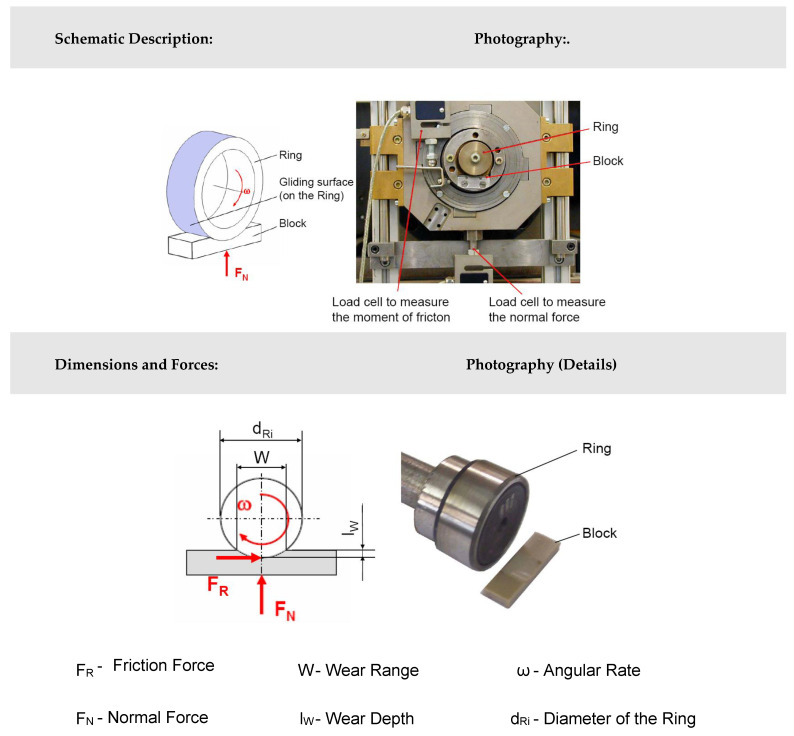
Standard test method for measuring resistance of plastics to sliding wear in block-on-ring wear test.

**Figure 5:**
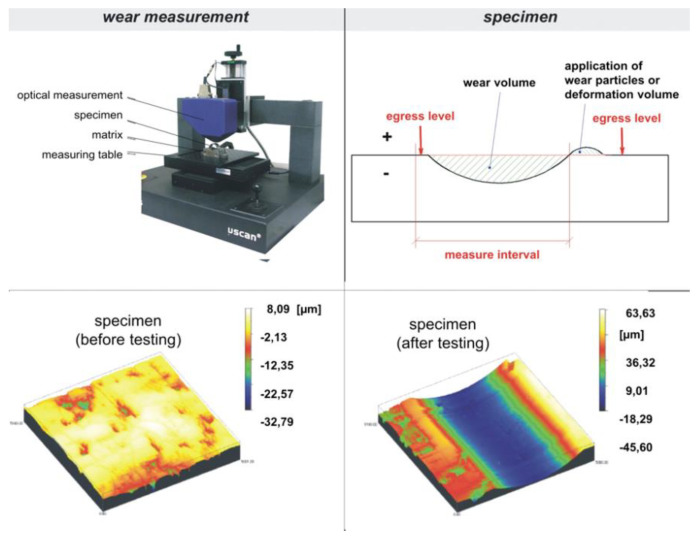
Optical wear measurement used in the study.

The modified block-on-ring test (Figure 4) is used to perform the tribological experiments in this work. The test rig is characterized by a polymer block that is pressed with a rotating hardened polished 100Cr6 steel shaft and diamond-like-coated (DLC) steel shaft. The test samples are mounted on an aluminum carrier plate with a high-strength adhesive to prevent the rigid body motion of the test piece. The volume loss of the block is recorded after the wear test. Additionally, the test rig is able to perform in situ measurements of the normal force, the coefficient of friction, and the temperature. Compared to other test procedures (cumulative wear method, DIN ISO 7148-2, ASTM G176-03), the major advantage of the developed setup is the simplicity of the specimens that are used. The identification of the specific wear rate is based on the comparative determination of the specimen before and after testing with an optical microscope (Figure 5). Special in-house software has been additionally used to evaluate the tribological parameters (the software operation is discussed in [34]). A variable-resistance transducer measures the moment of friction. The force of friction is obtained by multiplying the moment of friction with shaft radius (radius of steel or DLC shaft). The normal force is 100 N, the test duration is 10 h, and the sliding distance is approximately 10,000 m. The linear wear *W_l_* and the measured wear volume *Wv* are related to the sliding distance *s* by introducing the linear wear rate *w_l/s_* and the volumetric wear rate *w_V/s_*, respectively:

(1) $$w_{l/s}=\frac{W_{l}}{s}$$

(2) $$w_{V/s}=\frac{W_{V}}{s}$$

To incorporate the influence of the applied normal force *F_N_*, the specific wear rate *k* is defined as:

(3) $$k=\frac{W_{V}}{F_{N}\cdot S}=\frac{W_{V/s}}{F_{N}}$$

## 3. Result and Discussion

### 3.1. Tribological Results

Tribological examination of the composites is carried out to evaluate the coefficient of friction and specific wear rate against a 100Cr6 steel shaft (Rockwell Hardness on C scale - HRC 56, R_z_ 3.2 μm) and the DLC-coated 100Cr6 shaft (HRC 60, R_z_ 2.2 μm). The test configuration is as per the DIN ISO 7148, as described in Section 2.2.

**Figure 6:**
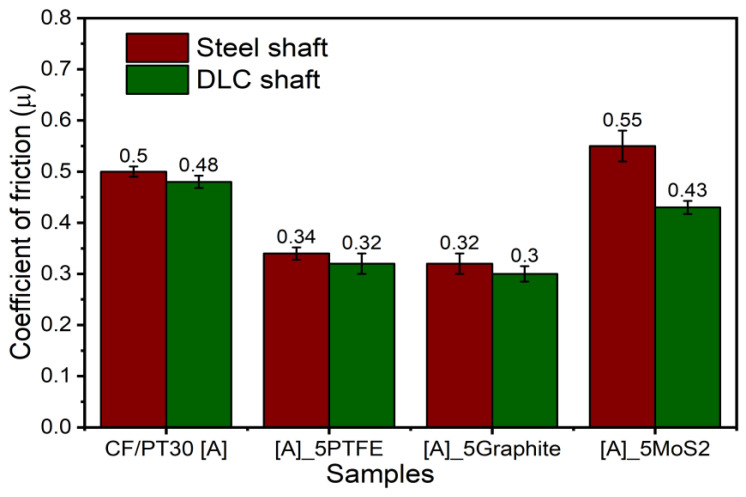
Coefficient of friction for different samples against steel and diamond-like-coated (DLC) shaft.

**Figure 7:**
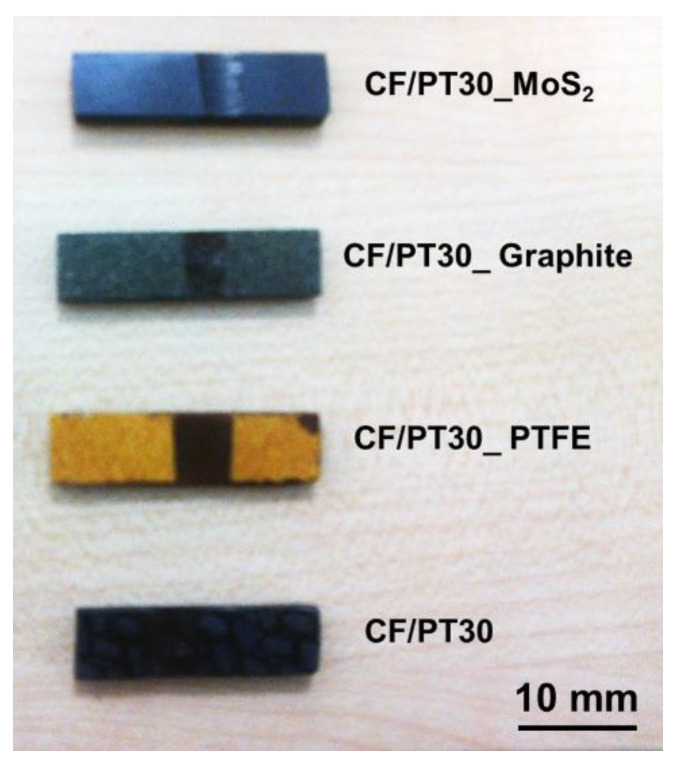
The block-on-ring samples after the tribological tests with steel shaft.

**Figure 8:**
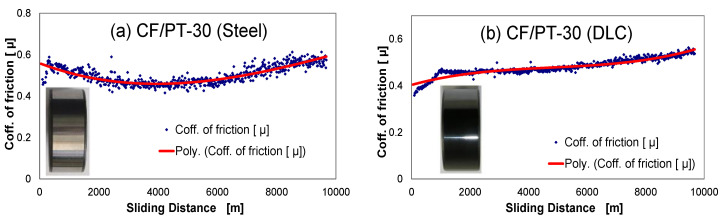

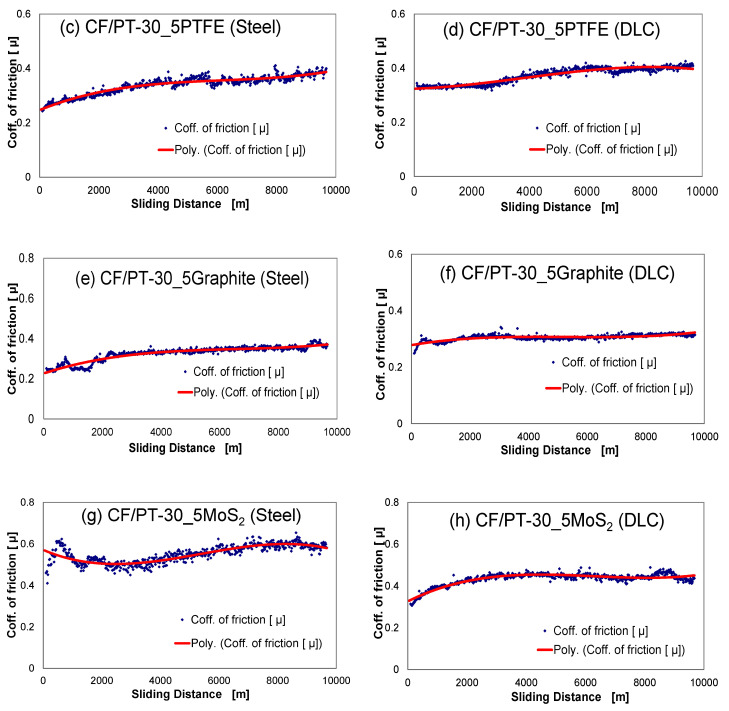
Coefficient of friction against sliding distance for different systems with steel shaft and DLC shaft. (**a**) CF/PT-30 (Steel), (**b**) CF/PT-30 (DLC), (**c**) CF/PT-30_5PTFE (Steel), (**d**) CF/PT-30_5PTFE (DLC), (**e**) CF/PT-30_5Graphite (Steel), (**f**) CF/PT-30_5Graphite (DLC), (**g**) CF/PT-30_5MoS_2_ (Steel), and (**h**) CF/PT-30_5MoS_2_ (DLC).

Figure 6 shows the determined values for coefficients of friction and the wear characteristic for the CF-reinforced CE polymers with 100Cr6 and DLC 100Cr6 as the tribological counter-face. The specific wear rate is relatively high. The DLC-hardened steel ring shows lower wear resistance under the unlubricated running condition due to the abrasive action of the fibrous wear particles. During the test, the counter-layer formed on the steel shaft, and these layers influence the running-in process, but it was determined that this process is limited to a small test period. Figure 7 shows different composite samples after the tribological tests. The curve of the coefficient of friction vs. sliding distance (Figure 8) at least shows no significant change concerning different running-in characteristics. The µ_avg_ value is the average coefficient of friction of three parallel trials on each specimen. The µ_avg_ for the unmodified PT-30 resin reinforced with the CF composite is observed to be 0.5 and 0.48 against the 100Cr6 steel shaft and DLC 100Cr6 shaft, respectively. The high value of the coefficient of friction is the reason why vigorous attempts are being made to modify the friction properties, to take advantage of otherwise superior mechanical and thermal properties of PT 30 resin. 

PTFE shears readily under the shear stress and is, therefore, the preferred material for the upper tribologically optimized layer in multilayered bearings. Its effectiveness is also evident from the reduction in friction in the tests. For the PT-30/CF/5 wt.% PTFE plate, µ_avg_ is reduced to 0.34 and 0.32 against the 100Cr6 shaft and DLC 100Cr6 shaft, respectively. Similarly, for the PT-30/CF/5 wt.% graphite plate, µ_avg_ is found to be 0.32 and 0.30 against the 100Cr6 shaft and the DLC 100Cr6 shaft, respectively. No significant improvement in the µ_avg_ is reported for the PT-30/CF/5 wt.% MoS_2_ plate. The µ_avg_ value is found to be very similar to the unmodified PT-30 resin reinforced with CF composite. The µ_avg_ is reported to be 0.55 and 0.43 against the 100Cr6 shaft and the DLC 100Cr6 shaft, respectively.

Reduction in the µ_avg_ value is attributed to self-lubricating properties of the additives and the formation of transfer films between the counter-faces. The coefficient of friction values drop slightly against the DLC 100Cr6 shaft, due to that fact that DLC-coated steel offers better hardness properties and is resistant to adhesive and abrasive wear. Due to different molecular structures of the used solid lubricants, the characteristics of transfer films are different. A similar trend in the friction behavior is also reported by [35]. 

### 3.2. Specific Wear Rate Results

**Figure 9:**
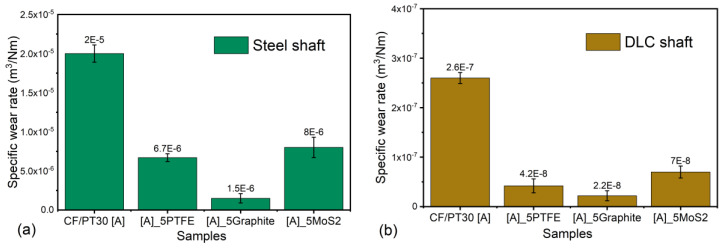
Specific wear rate values for different samples against (**a**) steel shaft and (**b**) DLC shaft.

**Figure 10:**
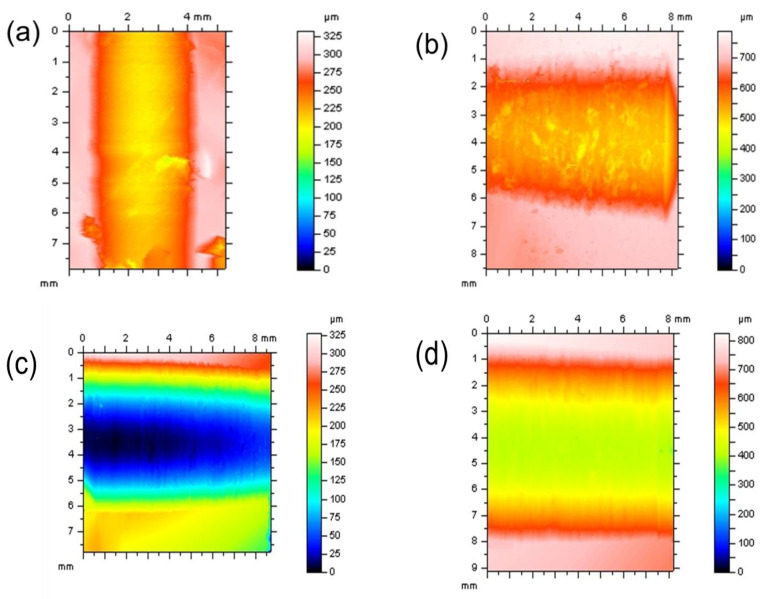
D profile of the wear track obtained in different composites: (**a**) CF/PT-30, (**b**) CF/PT-30 5PTFE, (**c**) CF/PT-30_5Graphite, and (**d**) CF/PT-30 5MoS_2._

The worn composite samples are examined for the volume loss of material and the specific wear coefficient. The specific wear rate is evaluated as discussed in Section 2.2. Figure 9 shows the results for the specific wear rate values for the four different composite specimens against the 100Cr6 shaft and the DLC 100Cr6 shaft, respectively. An average of the four samples is calculated and the error bars are shown. The specific wear rate for the unmodified PT-30 resin reinforced with the CF composite is computed to be 2 × 10^−5^ and 2.6 × 10^−7^ (m^3^/Nm) against the 100Cr6 shaft and the DLC 100Cr6 shaft, respectively. The specific wear rate decreases with the addition of PTFE. The specific wear rate for the PT-30/CF/5 wt.% PTFE plate is evaluated to be 6.7 × 10^−6^ k and 4.2 × 10^−8^ k (m^3^/Nm) against the 100Cr6 shaft and the DLC 100Cr6 shaft, respectively. Figure 10 shows the 3D profilometry of the samples used to calculate the wear loss.

Thus, the wear rate is reduced by 66% and 84% against the 100Cr6 shaft and DLC 100Cr6 shaft, respectively. The addition of the graphite filler further reduced the specific wear rate by 92% and 91% against the steel shaft and DLC shaft, respectively. The reported values of the specific wear rate against the shafts are 1.5 × 10^−6^ k and 2.2 × 10^−8^ k (m^3^/Nm), respectively. The addition of the MoS_2_ filler was found effective in bringing down the specific wear rate. A decrease of 60% and 73% is observed from the unmodified PT-30 resin reinforced with CF composite against the 100Cr6 shaft and DLC 100Cr6 shaft, respectively. The evaluated values of the specific wear rate are 8 × 10^−6^ and 7 × 10^−8^ (m^3^/Nm), respectively, for the two shafts.

The difference in the specific wear rate against the two different types of steel is due to the difference in the specific topography properties of the counterparts, as also explained by [35]. The DLC-coated shaft offers a much higher wear resistance compared to the steel shaft, thereby suppressing the abrasive wear, which causes a higher specific wear rate against the steel counterpart. The reduction in the specific wear rate within the four different composites for a particular composite–shaft pair is due to the self-lubricating properties of the fillers within the composites and due to transfer film formation between the counter-surfaces. 

### 3.3. Mechanical Results

**Figure 11:**
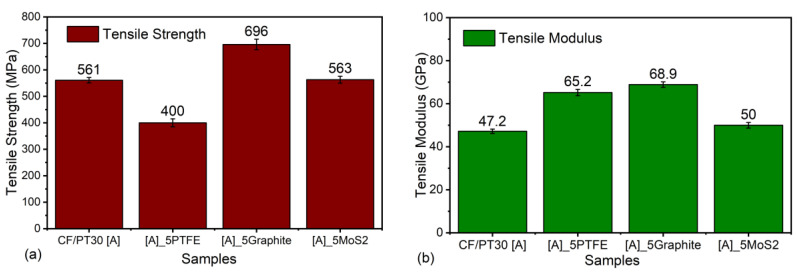
Comparison of (**a**) tensile strength of different composites and (**b**) tensile modulus of different composites.

The tensile strength and tensile modulus are also identified for four different composites. Ten specimens from each composite are tested to obtain an average value. The results are shown in Figure 11. For the unmodified PT-30 resin reinforced with a CF, a tensile modulus of 47 GPa and tensile strength of 561 MPa are measured. For all other composites modified with fillers, the modulus value is higher than that of the reference system. On the other hand, when the tensile strength is compared, other systems have similar or higher values of tensile strength except PTFE-based systems. The highest value of tensile strength is achieved for the graphite-based system, which is approximately 25% higher than the reference system, which may possibly be due to the strong adhesion between fibers, graphite fillers, and matrix. The inclusion of PTFE led to a decrease in the tensile modulus and tensile strength, as indicated by lower values, which may be due to the deterioration of PTFE microparticles at elevated curing; these results are further supported by the finding of Bijwe et al. [36] who also reported the same kind of deterioration of mechanical properties in their work. The addition of MoS_2_ does not show any significant improvement in the tensile strength, as well as in the elastic modulus of the material.

## 4. Conclusions

CE thermosetting resins are attractive due to their wide range of mechanical and thermal properties. However, due to the brittle nature, their industrial applications as a friction and wear material are limited. This paper proposes various techniques for the improvement in tribo-mechanical properties of the CE resins by utilizing filler materials. CE resins-based carbon fiber-reinforced composites are infiltrated with graphite, PTFE, and MoS_2_ fillers and manufactured using the RTM method. The mechanical strength and tribo-properties are analyzed, and the results are reported in this work. It is observed that out of all the fillers used, graphite proved to be a better candidate, offering a lower specific wear rate, a lower friction value, and a higher tensile strength and tensile modulus as compared to the reference system. The obtained results can be used to manufacture maintenance-free slider bearings, which can be used continuously at a service temperature of 300 °C with good tribo-mechanical properties.

## Figures and Tables

**Table 1:** Friction coefficients for some common materials against different materials [33]

Material	Counter-Face Material	Dry Contact Static Friction (µ)
Aluminum	Aluminum	1.10–1.35
Aluminum	Steel	0.61
Brake (composite)	Cast iron	0.40
Brass	Steel	0.50
Bronze	Cast iron	0.21
Copper	Steel	0.53
Diamond	Steel	0.10
Graphite	Steel	0.10
Polyethene	Steel	0.2
Polystyrene	Steel	0.30–0.35
PTFE (Teflon)	Steel	0.04
Epoxy resin	Steel	0.71
Cyanate ester resin	Steel	0.50
Bismaleimide resin	Steel	0.65

**Table 2:** Properties of cyanate ester resin (PT-30) from supplier data.

Property	Values
Tensile strength [GPa]	0.041
Tensile modulus [GPa]	4.07
Compressive strength [GPa]	0.317
Elongation	1.2–1.5
Gel time at 200°C [min]	30–70
Viscosity at 80°C [mPa*s]	300–500
